# Integration of a Return-to-Work Module in Cognitive Behavioral Therapy in Patients With Major Depressive Disorder and Long-Term Sick Leave—A Feasibility Study

**DOI:** 10.3389/fpsyt.2020.00512

**Published:** 2020-06-03

**Authors:** Lotta Winter, Julia Geldmacher, Katharina Plücker-Boss, Kai G. Kahl

**Affiliations:** Department of Psychiatry, Social Psychiatry and Psychotherapy, Hannover Medical School, Hannover, Germany

**Keywords:** return to work, major depressive disorder, feasibility, acceptability, work ability

## Abstract

**Objective:**

Major depressive disorder (MDD) has a negative impact on individuals ability to work, and is often associated with long phases of sick leave. Consequently, interventions facilitating return to work in patients with MDD gained increased attention during last decades. We here report results of a feasibility study where a “return-to-work” (RTW) module published by Lagerveld and colleagues in the Netherlands was integrated in cognitive behavioral therapy in depressed patients with long-term sick leaves in Germany. Our study aimed to answer the following questions: Is RTW accepted by patients and therapists? Do RTW interventions lead to return-to-work? Do depressive symptoms improve?

**Methods:**

Twenty patients with MDD (15 female; mean age, 45 ± 9 years) were included. Patients received cognitive-behavioral therapy with an integrated, standardized return-to-work module (W-CBT). Psychometric measurements included Beck Depression Inventory (BDI-2) and work ability index (WAI). Further, time until return-to-work was measured, and acceptability of W-CBT was assessed using visual analog scales and open questions.

**Results:**

Mean sick leave days in depressed patients were 127 ± 97, and 75% of patients were sick leave for more than 6 weeks. After treatment, 11/20 patients had returned to their former occupation (55%), 5/20 were in occupational re-deployment or started a new job (25%), and 3/20 patients were still on sick leave (2/20; 10%) or received a pension (1/20; 5%). One patient dropped out. BDI-2 sum score improved from 23 ± 8 to 8 ± 5 (p < 0.001), and WAI improved from 28 ± 6 to 39 ± 7 (p < 0.001). Acceptability of W-CBT in patients and therapists was high.

**Conclusion:**

We here demonstrate feasibility and acceptability of an RTW module integrated in standard cognitive behavioral therapy. W-CBT leads to improvement of work ability, paralleled by improvement of depressive symptoms. Despite the limitations of this uncontrolled study, the results propose that W-CBT may be feasible in the treatment of depressed patients with long sick leaves and justify a controlled trial evaluating the efficacy of W-CBT.

## Introduction

For the majority of people work is an important part in life. Work meets important psychosocial needs, is central to individual identity, and social role and employment is one of the main drivers of social gradients in physical and mental health and mortality (1). Decreased work participation due to mental illness has increased considerably in western countries (2). Associated with impaired work functioning and problems in work participation such as long-term sick leave are, e.g., depression, anxiety, and adjustment disorder (3, 4). Decreased work participation leads to an additional burden for the patient, fostering social isolation, and enhancing the risk for early retirement since only around 50% of people who are on long-term sick leave return into their occupation (5, 6). This indicates a risk of entering disability due to mental illnesses. Variables like symptom severity or comorbidity rate as part of illness characteristics explain part of disability processes, but contextual factors (personal and environmental factors like days out of role or work absence) also play an important role in the disablement process (7). For people suffering from depression several studies have shown increased difficulties to go back to their workplace or to keep their position when returning to work after depression-related unemployment or sick leave (8–10). Even the risk for further relapses of depression can be influenced by experiencing difficulties when going back to work (8). In a recent study Weziak-Bialowolska et al. (11) found that depression in life is likely to manifest itself at work. As listed above work participation provides health-promoting effects. Facilitating return to work and reducing the number of sick leave days is an objective shared by patients, companies, and the public health system.

Sick leave episodes in patients with mental illness are characterized by long duration and high frequency (12). In general, sick leave duration depends on several factors such as medical (e.g., diagnosis, presence of somatization, severity, comorbidity) (2, 5, 13), job-related (e.g., occupational stress, threat of unemployment, organizational structure) (5), socio-demographic (e.g., age, gender, educational level, marital status) (14), legislative and administrative factors (e.g., modality for paying) (15). However, studies indicate that an important factor in sick leave duration in people with mental disorders is delay in their management (16). The delay in treating patients with mental disorders may extend the time being on sick leave. Studies carried out with mentally ill workers found that a delay of more than 3 weeks in the medical consultation after going on sick leave was one of the main factors predicting a long sick leave of between 3 to 6 months (2). Besides reducing the time between beginning and diagnosis/treatment of mental disorders, integration of return to work aspects into treatment may facilitate occupational participation and reduce sick leave days due to mental disorders (17). In a recent study by Lagerveld and colleagues, a work-focused cognitive behavior therapy (W-CBT) in patients with minor mental illness (primarily adjustment disorder) was superior in terms of return to work compared to cognitive behavior therapy (CBT) alone. These results suggested that focusing more and earlier on work-related aspects in patients with mental disorder improved occupational participation within a regular psychotherapeutic setting, without negative side effects on psychological complaints (17). The “return-to-work” (RTW) module used in this study was developed by Lagerveld and colleagues (from Netherlands) to be integrated in standard CBT. This RTW module was translated into German language and published recently (18).

The aim of our study was to examine whether it is feasible to integrate the translated RTW module into CBT in Germany in the treatment of patients with major depressive disorder (MDD) who are on sick leave due to their mental illness. We aimed to examine whether W-CBT is accepted by patients and therapists, whether patients return to work, and whether reduction of depressive symptoms is impeded.

## Methods

For the first time an authorized translated version of the original RTW module developed in the Netherlands was applied by RTW-trained psychotherapists in Germany. The study was approved by the ethics committee of the Hannover Medical School, and all participants gave informed written consent prior to the beginning of the study. The psychotherapeutic intervention followed ethical guidelines for interventional studies according to the declaration of Helsinki.

### Participants and Procedure

Inclusion criteria were the diagnosis of MDD, sick leave due to MDD, age >18 years, and outpatient treatment. Exclusion criteria were any substance-use disorder, bipolar disorder, any personality disorder, any eating disorder, and any acute or chronic physical disorder. During intake at the outpatient clinic, patients were screened for inclusion criteria. Further, acute suicidality (severe suicidal thoughts, suicide intention) was an exclusion criterion. All patients who fit criteria and agreed to be included into the study underwent a structured clinical interview according to DSM-IV (SCID-1 and SCID-2), and a thorough clinical examination to confirm in- and exclusion criteria. None of the patients who were invited refused to take part in the study.

Psychological assessments further comprised the Beck Depression Inventory-2 (BDI-2), and the work ability index (WAI). **Table 1** presents demographic information of the patients. The participants were asked to fill in the BDI-2 and the WAI before treatment started (T0), and after the last treatment session (T1). In addition, at T1 the patients and therapists were asked to reply to qualitative questions about the therapy process in written form. Patients received these questions by their therapists after the last psychotherapeutic session (T1), and the psychotherapists received the qualitative questions by the study leader after the last treatment session. These questions are listed in **Table 2**.

**Table 1:** Baseline data of depressed patients included in the study.

	Patients with major depressive disorder (N= 20)
Female gender (N/%)	15 (75%)
Age (y)	45.8 ± 9.5
Mean BDI-2 sum score before treatment	23.6 ± 8.5
Marital status (N/%)	
Singled	4 (20%)
Partnered	6 (30%)
Married	7 (35%)
Divorced	3 (15%)
Average sick leave until study entry (d)	127 ± 97
Long-term sick leave (>6 week) (N/%)	15 (75%)
Graduation/schoolyears (N/%)	
Secondary modern school (5–9 years)	3 (15%)
Junior high school (10–11 years)	7 (35%)
Upper school (12–13 years)	10 (50%)
Occupation	
Blue collar	11 (55%)
White collar	9 (45%)

**Table 2:** List of qualitative questions addressed to the patients and therapists.

Questions to the patients:	
1	How satisfied were you overall with your therapy? (VAS 0–15)
1.1	The following approaches in therapy have helped me a lot:
1.2	The following approaches in therapy have helped me less:
2	How much did the therapy support returning to work? (VAS 0–15)
2.2	What helped you in therapy with resuming your work?
2.3	Which aspects of the therapy have hindered the resumption of work?
3	How much did you feel supported outside the therapy (resettlement officer, doctor, supervisor, colleagues, etc.) in resuming work? (VAS 0–15)
3.1	What or who else helped you get back to work outside of therapy?
3.2	What or who has hindered the return to work?
4	How helpful did you think it was that the focus of the therapy was placed on the workplace and on professional reintegration? (VAS 0–15)
**Questions to the therapists**	
1	How helpful did you think it was that the focus of the therapy was placed on the workplace? (VAS 0–15)
2	Do you think the patient benefited from the RTW approach? (VAS 0–15)
2.1	What did the patient most benefit from?
2.2	Did the RTW approach in any way hinder the patient’s recovery?
3	As a therapist, do you feel that the RTW approach has helped you in any way to treat this patient? (VAS 0–15) (Please describe.)
4	Despite the module, were there any obstacles in the reintegration of your patient? (Please describe.)

All participating psychotherapists were trained in cognitive behavioral therapy and had at least 3 years of working experience in psychotherapy in the Department of Psychiatry, Social Psychiatry and Psychotherapy. They were trained in the “return-to-work” (RTW) module during a weekend course which was held by the inventors of the manual (SE Lagerveldt, RWB Blonk), and the psychotherapeutic process was continuously supervised.

### Description of the Interventions in W-CBT

In short, W-CBT offers a manualized stepwise program that focusses on the process of returning to work from the first session on. Workplace and work-related issues are set in the center of the therapy, and classical CBT strategies, such as socratic dialogue, behavioral experiments, or disputation of dysfunctional thoughts, are used.

After assessment and diagnostics regular CBT was prepared. This included setting individual therapy goals and developing a treatment plan. Based on the “Return-to-work” protocol by Lagerveld and Blonk (9) the general treatment plan was supplemented by the manualized RTW elements (W-CBT). Following the protocol the psychotherapist conducted the process of returning to work. Therapists focused on work issues from the first session on and used work as a context to reach treatment goals (e.g., activation, time structure, social contact, regular activity, increasing self-esteem). Therefore, detailed information on the individual’s work history as well as on the conditions and procedures at the workplace needed to be collected. Communication and cooperation with fellow-treating parties is obligatory. In the first session all issues were listed, and their relation to work was illustrated (e.g., “You said you often experience listlessness, in which situations do you notice it at work?”), information about the relationship between psychological well-being and work was shared (e.g., “Work can offer structure, social contacts, and self-esteem that are important for psychological well-being.”) and for homework the patient prepared a detailed description of the conditions at the workplace. In the second session, the conditions at work got analyzed. Despite collecting information on the framework conditions all tasks were listed and ranked in a hierarchy comparable to an “anxiety hierarchy” used in exposure *in vivo* techniques. As homework the patient was motivated to contact the person in charge for reintegration at the company. In the third session to prepare occupational reintegration all generated information were used to develop a schedule for the reintegration process. Therefore, the needed period got estimated, the first step in its scope and schedule was planned as well as difficulties needed to be identified. The plan for the gradual reintegration process considered the activities or tasks that the patient would perform, the time that was spent on each task and whether modifications on workplace conditions were necessary. Once the patient had started with the reintegration schedule part of the therapy sessions were used to evaluate the process and adjust the schedule if necessary. The therapist supported the full procedure and new skills, alternative behavior, cognitive restructuring, etc. was applied to the examples confrontation with work generated. The German translation of the manual was followed (10). Therapy was finished when individual goals were achieved, and both therapists and patient agreed on ending the treatment. Supervisors trained by Lagerveld supervised the therapy processes on a regular basis.

### Data Analysis

All statistical analyses were conducted using SPSS version 25. Descriptive analysis was performed for the whole group concerning age, gender, marital status, graduation, average days on sick leave, and visual analog scales of the qualitative questions. The responses to all other qualitative questions (open answer questions) were listed in the results.

Pre-post comparisons for the Beck Depression Inventory-2 (BDI-2) BDI sum score and for the WAI were performed with paired t-test.

To compare patients who successfully returned to work or were in occupational redeployment/new job (N = 16) with those who did not (early pension, N = 1; still on sick leave, N = 2) or dropped out (N = 1), baseline depression, and work-ability data were compared using Mann-Whitney U-test.

## Results

### Is W-CBT Accepted by Patients and Therapists? Results of the Qualitative Questions

Out of 20 patients, only one dropped out during W-CBT treatment as he moved to a different city. To assess acceptability of W-CBT, patients and psychotherapists were asked to answer a qualitative questions after the last W-CBT session (after an average of 26 ± 14 weeks).

**Figure 1:**
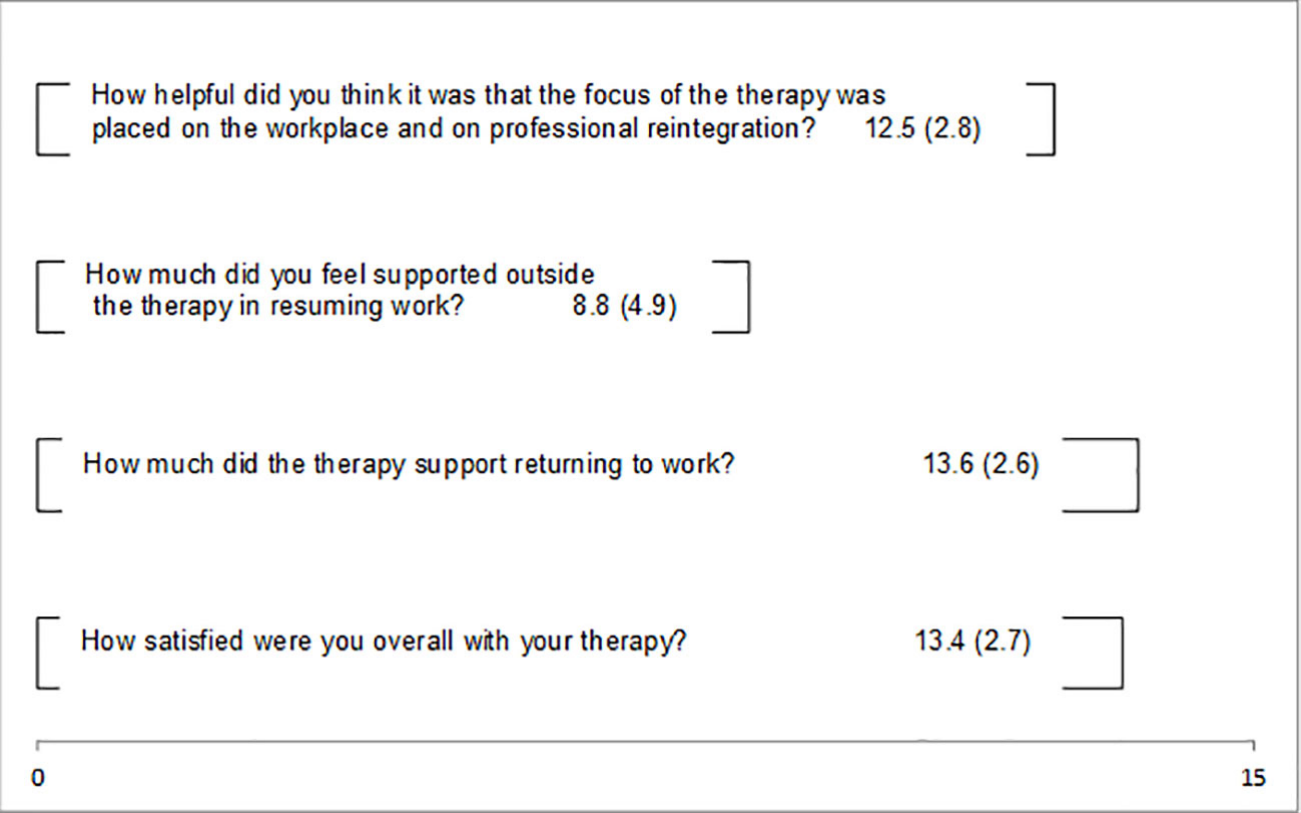
Results of the acceptability questionnaire in patients.

**Figure 2:**
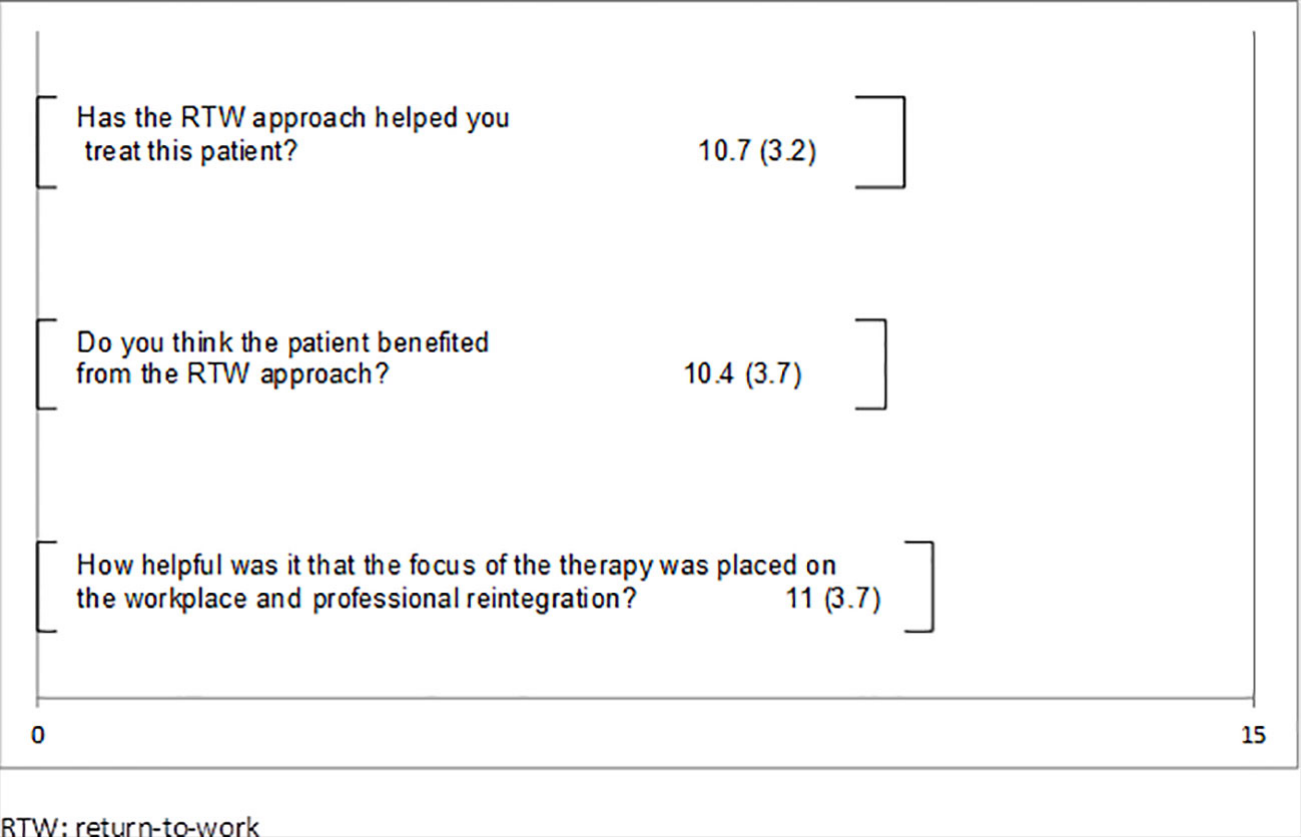
Results of the acceptability questionnaire in therapists.

The patients could answer four of the qualitative questions with the use of 15 cm long visual analog scales ranging from “not at all” (11) to “very much” (15). The means and standard deviations are presented in **Figure 1**. Between the two poles all means lie above the middle presenting positive ratings. The patients experienced the focus on work as helpful (M = 12.5), supportive in the process of returning to work (M = 13.6), and were generally satisfied with their therapies (M = 13.4). The support outside therapy to resume to work was scored the lowest (M = 8.8). **Figure 2** shows the therapist’s feedback on three visual analog scales. These means also lie above the middle towards the positive pole. Therapists evaluated the approach as helpful for the treatment (M = 10.7), believed the patients benefited from the approach (M = 10.4), and rated the work focus as helpful (M = 11).

To the question what helped you a lot during therapy patients replied: “having someone to talk about my challenges,” “behavioral analysis,” “following a weekly schedule,” “developing alternative strategies and alternative appraisals,” “learning to take more care of myself and my needs,” “preparing difficult situations in role plays.” The only answer given to the question what was less helpful was: “filling out questionnaires.” What helped in therapy with resuming work was “to gain clarity on decisions,” “role plays,” “to feel prepared knowing new strategies,” “learning how I can influence the situation at work.” No answers were given to the question what aspects hindered the reintegration to work. Different family members were listed as people who have supported the process of reintegration to work. In addition some patients mentioned the representative for employees with disabilities, three patients listed their direct superiors, some listed their colleagues and some the company doctor. Seven patients reported that their direct supervisor hindered the resumption to work, two listed their colleagues. To the question “What did the patient most benefit from?” the therapist answered: “support and motivation to communicate with the employer,” “developing a precise schedule for reintegration,” “job analysis,” “role plays,” “precise analysis of obstacles at work,” “the structured approach,” “situation analysis,” “changing beliefs.” The question if the RTW-module in any way hindered the therapy process was consistently answered with “no.” Aspects that helped the therapists in the treatment of the patients were “being obligated to focus on work from the first session on helped doing it and hindered avoiding it,” “it helped keeping the focus on work even if the patient tried to avoid it,” “it created a helpful structure even in the treatment of severe symptoms of depression,” “the detailed information on the workplace prevented generalized assumptions,” “the detailed information on the workplace helped to estimate chances and challenges,” “the module helped me to feel more secure in the support of the reintegration process.” As obstacles in the reintegration process, the therapists described that in five cases, the employer did not cooperate, e.g., terminated the job contracts or offered lower positions than expected. From one therapy the therapist reported that due to severe chronic depression with a comorbid personality disorder the approach was too demanding. In two cases the patients did not follow the plan, but tried to be faster. This led to overstress. In some cases bureaucracy was reported to be very slow which delayed the reintegration process.

### Do RTW Interventions Lead to Return-to-Work?

**Figure 3:**
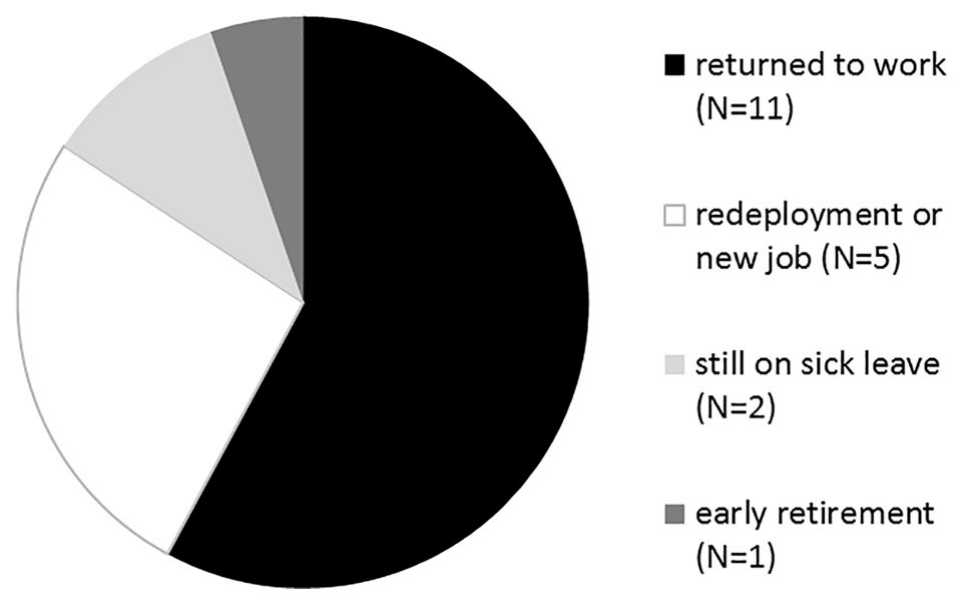
Treatment outcome concerning return to work.

After treatment, 11/20 patients had returned to their former occupation (55%), 5/20 were in occupational re-deployment or had started a new job (25%), and 3/20 (15%) patients were still on sick leave (2/20; 10%) or received a pension (1/20; 5%) (shown in **Figure 3**). Number of therapy sessions: 26 (± 14), sessions until RTW: 9 (± 9).

We found higher depression sum scores (31.5 ± 12.7 versus 23.1 ± 8.9) and lower work ability scores (23.2 ± 5.7 versus 28.5 ± 6.2) at baseline in those patients who failed to return to work. However, these differences did not reach significance.

### Do Depressive Symptoms and Work Ability Improve?

**Figure 4:**
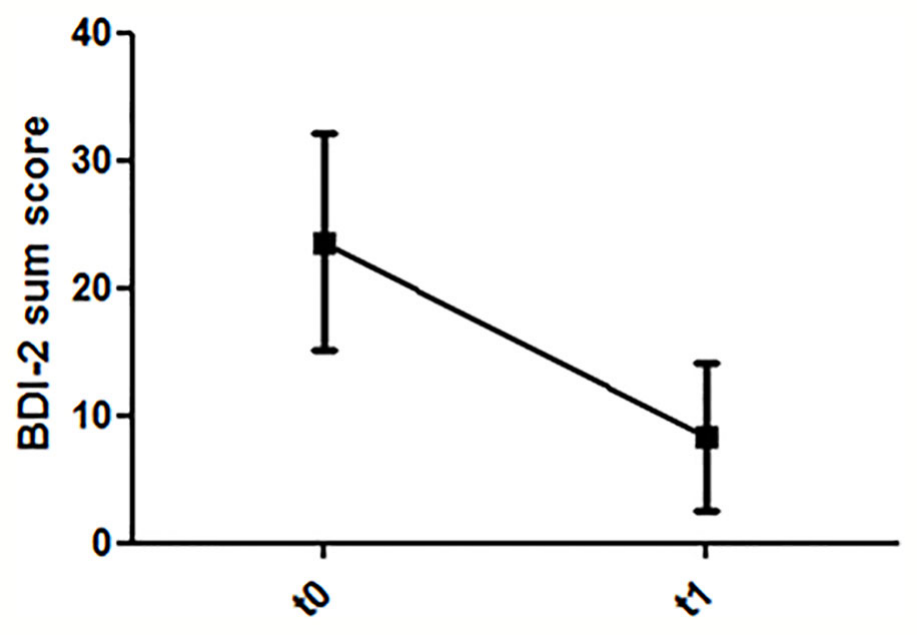
Dynamics of depression ratings (T0: before first treatment session; T1: after last treatment session).

**Figure 5:**
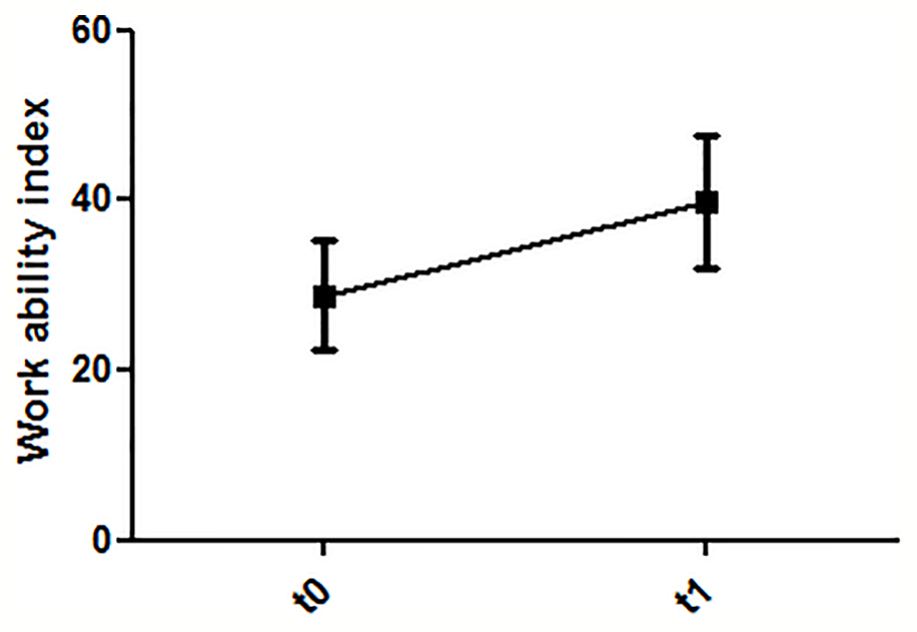
Dynamics of work ability (T0: before first treatment session; T1: after last treatment session).

Pre-post comparisons of 19 patients revealed a reduction of depressive symptoms (T = 6.7; df = 18; p < 0.001) (**Figure 4**). The sum scores of the WAI showed a significant improvement of the patient’s work ability in the pre-post comparison (T = 7.8; df = 18; p < 0.001) (**Figure 5**).

## Discussion

The main results of our study are: RTW module could successfully be integrated in CBT, and was highly accepted by patients and psychotherapists. Further, the majority of patients with MDD treated with W-CBT started occupational reintegration during treatment, or started occupational re-deployment/working in a new job. Depressive symptom severity was reduced, and work ability significantly increased.

Our results extend the existing literature on W-CBT. First, we included only patients with major mental disorder, i.e., MDD, with moderate symptom severity. In the initial study by Lagerveld and colleagues, mainly patients with minor mental disorders, i.e., adjustment disorder, were included, leaving the question open whether W-CBT could also be applied to patients with major depression (17).

In a recent study by Kröger and colleagues, a work-related CBT treatment was offered to 13 patients with MDD and compared to a matched control group (19). They found that days of incapacity to work were reduced in both groups, although employees in the work-related CBT group showed fewer days of incapacity to work, and significantly more employees were working in the work-related CBT group at 1 year follow-up (13/13 versus 8/13 in the control group). Depression severity scores were similarly reduced in both groups, leading to response (defined as 50% symptom reduction) in both groups at the end of treatment, and at 1 year follow-up. In this study, the majority of patients was mild-to-moderately depressed with a mean BDI sum score of 20 in both groups, and only short-term sick leaved patients (maximum 3 weeks sick leave) were included. Psychotherapists in this study received a 2-h workshop in which aims and procedures of work-related treatment were introduced. Measures of acceptability by patients and therapists were not assessed.

Our results demonstrate that W-CBT can also be integrated in the psychotherapeutic treatment of patients with more severe disorder and considerably longer sickness leave. In regular CBT offered by mental health professionals a focus on return to work is often lacking (20). The effectiveness of psychotherapy on return to work may therefore be enhanced when work is more explicitly addressed during treatment. The work-focused CBT intervention employs the same conceptual framework as is used for regular CBT, based in large on the work by Beck (21). In short, CBT states that dysfunctional behavior and mental health symptoms are not merely the consequence of a stressful situation (e.g., work pressure), but that appraisal of this situation plays a crucial role (21). Two main intervention approaches can be distinguished, targeting dysfunctional cognitions, and acquiring effective skills such as behavioral activation.

The central idea in work-focused CBT interventions is that any CBT technique may be applied to the work context, in order to achieve regular psychotherapy treatment goals (improvement in depressive symptoms) and to facilitate return to work (17). Several factors may impede return to work in patients with MDD, such as feelings of incompetency, feelings of excessive demands, lack of motivation, or lack of energy. CBT techniques can be applied to relieve feelings of incompetency, to change challenging/stressful work situations, to change dysfunctional avoidance behavior related to the workplace (such as procrastination), or to change the appraisal of work-related stressors (17). Hence, it is assumed that integrating return to work aspects in CBT would contribute to a change in dysfunctional work-related cognitions and behaviors, thereby facilitating return to work in MDD patients.

As from clinical experience we know that many patients come into treatment with the idea never being able returning to work as well as many therapists share the opinion that it may be beneficial for the patient to have a break from the stressful environment and have time to recover before confronting work issues we were interested in the acceptability of the RTW-module. The results show a high acceptability. Not all of the patients initially agreed to focus on work and reintegration to work, but by sharing information on the relationship between work and how it can support recovering from mental disorders, e.g., by providing structure, social contacts, opportunities to experience competence, and self-esteem, all patient became interested in the procedure. Not a single answer was given to the question if the RTW-module hindered the therapy process. No negative feedback could be found even though there was a certain level of skepticism in the beginning.

A potential concern against W-CBT might be that patients and therapists think that early return to work may increase relapse or worsen depressive symptoms. However, we found in our study that patients who successfully returned to work had responded to psychotherapeutic treatment, indicated by a >50% reduction in self-rated depressive symptoms. We conclude that W-CBT does not impede recovery from depressive symptoms in our patient sample. Of note, those patients who failed to return to work were more severely ill with a mean BDI sum score (on average 31.5) pointing to severe MDD. Further, these patients had lower work ability with a mean WAI score (on average 23.2) pointing critically reduced work ability. In contrast, patients who successfully returned to work, were in occupational redeployment or starting in a new job, had moderate depression severity with an average BDI sum score of 23.1, and a better work ability (28.5 on average) pointing to a moderate reduction. Although the study group is too small to draw firm conclusions from this observation, one may speculate that W-CBT might be recommended rather in moderately ill patients with MDD, and in those with at least moderate work ability.

The main limitations of our study are: the small sample size and a missing control group. Further, we observed patients only for an average of 26 weeks. However, the principal aim of our study was to explore feasibility and patients and therapists acceptability. Therefore, future studies might take these limitations into account, using a greater sample size and a longer follow-up.

We conclude from our data that a Return-to-Work module can successfully be integrated in CBT in Germany, leading to work participation even in patients with long sick leave, and leading to a reduction of depressive symptoms. The focus on work from the start of psychotherapy on was well accepted by patients and therapists. However, due to the small number of participants the results of our feasibility study have to be interpreted carefully.

## Data Availability Statement

The datasets generated for this study are available on request to the corresponding author.

## Ethics Statement

The study was approved by the local ethics committee at the Hannover Medical School. All patients/ participants provided their written informed consent to participate in this study.

## Author Contributions

All authors put up the study design and were involved translating the protocol. LW, JG, and KP-B screened the patients and were part of the group of therapists. JG and KP-B were responsible for data collection. LW and KK supervised the therapies, analyzed the data and wrote the manuscript.

## Conflict of Interest

The authors declare that the research was conducted in the absence of any commercial or financial relationships that could be construed as a potential conflict of interest.

## References

[1] Waddell G Burton K Is Work Good for Your Health and Wellbeing? An Independent Evidence Review. London, UK: The Stationary Office (2006).

[2] Brouwers EP Terluin B Tiemens BG Verhaak PF Predicting return to work in employees sick-listed due to minor mental disorders. J Occup Rehabil (2009) 19:323–32. 10.1007/s10926-009-9198-8 PMC2775114 19760489

[3] Andrea H Bultmann U Beurskens AJ Swaen GM van Schayck CP Kant IJ Anxiety and depression in the working population using the HAD Scale, psychometrics, prevalence and relationships with psychosocial work characteristics. Soc Psychiatry Psychiatr Epidemiol (2004) 39:637–46. 10.1007/s00127-004-0797-6 15300374

[4] Boedeker W Klindworth H Hearts and minds at work in Europe. A European work-related public health report on cardiovascular diseases and mental ill health. Essen, Germany: BKK Bundesverband (2007).

[5] Blank L Peters J Pickvance S Wilford J Macdonald E A systematic review of the factors which predict return to work for people suffering episodes of poor mental health. J Occup Rehabil (2008) 18:27–34. 10.1007/s10926-008-9121-8 18213510

[6] Dekkers-Sanchez PM Hoving JL Sluiter JK Frings-Dresen MH Factors associated with long-term sick leave in sick-listed employees: a systematic review. Occup Envorin Med (2008) 65:153–7. 10.1136/oem.2007.034983 17881466

[7] van der Werff E Verboom CE Penninx BW Nolen WA Ormel J Explaining heterogeneity in disability associated with current major depressive disorder: effects of illness characteristics and comorbid mental disorders. J Affect Disord (2010) 127:203–10. 10.1016/j.jad.2010.05.024 20594596

[8] Lerner D Adler DA Chang H Lapitsky L Hood MY Perissinotto C Unemployment, job retention, and productivity loss among employees with depression. Psychiatr Serv (2004) 55:1371–8. 10.1176/appi.ps.55.12.1371 PMC4283817 15572564

[9] Lépine J-P Briley M The increasing burden of depression. Neuropsychiatr Dis Treat (2011) 7:3. 10.2147/NDT.S19617 21750622 PMC3131101

[10] Amos TB Tandon N Lefebvre P Pilon D Kamstra RL Pivneva I Direct and Indirect Cost Burden and Change of Employment Status in Treatment-Resistant Depression: A Matched-Cohort Study Using a US Commercial Claims Database. J Clin Psychiatry (2018) 79:1–9. 10.4088/JCP.17m11725 29474009

[11] Weziak-Bialowolska D Bialowolski P Sacco PL, VanderWeele TJ and McNeely E. Well-Being in Life and Well-Being at Work: Which Comes First? Evidence From a Longitudinal Study. Front Public Health (2020) 8:103. 10.3389/fpubh.2020.00103 32328472 PMC7160299

[12] van der Klink JJ Blonk RW Schene AH van Dijk FJ Reducing long term sickness absence by an activating intervention in adjustment disorders: a cluster randomised controlled design. Occup Environ Med (2003) 60:429–37. 10.1136/oem.60.6.429 PMC1740545 12771395

[13] Flach PA Groothoff JW Krol B Bultmann U Factors associated with first return to work and sick leave durations in workers with common mental disorders. Eur J Public Health (2012) 22:440–5. 10.1093/eurpub/ckr102 21840894

[14] Nieuwenhuijsen K Verbeek JH de Boer AG Blonk RW van Dijk FJ Predicting the duration of sickness absence for patients with common mental disorders in occupational health care. Scand J Work Environ Health (2006) 32:67–74. 10.5271/sjweh.978 16539174

[15] Krause N Frank JW Dasinger LK Sullivan TJ Sinclair SJ Determinants of duration of disability and return-to-work after work-related injury and illness: challenges for future research. Am J Ind Med (2001) 40:464–84. 10.1002/ajim.1116 11598995

[16] Burstrom B Nylen L Clayton S Whitehead M How equitable is vocational rehabilitation in Sweden? A review of evidence on the implementation of a national policy framework. Disab Rehabil (2011) 33:453–66. 10.3109/09638288.2010.493596 20528191

[17] Lagerveld SE Blonk RW Brenninkmeijer V Wijngaards-de Meij L Schaufeli WB Work-focused treatment of common mental disorders and return to work: a comparative outcome study. J Occup Health Psychol (2012) 17:220–34. 10.1037/a0027049 22308965

[18] Winter L Kraft J Boss K Kahl KG Return to Work: A Workplace Focused Module to be Integrated in Cognitive Behavioral Therapy. Psychother Psychsom Med Psychol (2015) 65:321–6. 10.1055/s-0035-1545312 25919056

[19] Kroger C Bode K Wunsch EM Kliem S Grocholewski A Finger F Work-related treatment for major depressive disorder and incapacity to work: preliminary findings of a controlled, matched study. J Occup Health Psychol (2015) 20:248–58. 10.1037/a0038341 25402222

[20] Rebergen DS Bruinvels DJ van Tulder MW van der Beek AJ van Mechelen W Cost-effectiveness of guideline-based care for workers with mental health problems. J Occup Environ Med (2009) 51:313–22. 10.1097/JOM.0b013e3181990d8e 19225416

[21] Beck AT Cognitive therapy and the emotional disorders. International Universities Press (1976).

